# Visualising biological data: a semantic approach to tool and database integration

**DOI:** 10.1186/1471-2105-10-S6-S19

**Published:** 2009-06-16

**Authors:** Steve Pettifer, David Thorne, Philip McDermott, James Marsh, Alice Villéger, Douglas B Kell, Teresa K Attwood

**Affiliations:** 1 School of Computer Science, University of Manchester, Manchester, M13 9PL, UK; 2 School of Chemistry, University of Manchester, Manchester, M13 9PL, UK; 3 Faculty of Life Sciences, University of Manchester, Manchester, M13 9PL, UK

## Abstract

**Motivation:**

In the biological sciences, the need to analyse vast amounts of information has become commonplace. Such large-scale analyses often involve drawing together data from a variety of different databases, held remotely on the internet or locally on in-house servers. Supporting these tasks are *ad hoc *collections of data-manipulation tools, scripting languages and visualisation software, which are often combined in arcane ways to create cumbersome systems that have been customised for a particular purpose, and are consequently not readily adaptable to other uses. For many day-to-day bioinformatics tasks, the sizes of current databases, and the scale of the analyses necessary, now demand increasing levels of automation; nevertheless, the unique experience and intuition of human researchers is still required to interpret the end results in any meaningful biological way. Putting humans in the loop requires tools to support real-time interaction with these vast and complex data-sets. Numerous tools do exist for this purpose, but many do not have optimal interfaces, most are effectively isolated from other tools and databases owing to incompatible data formats, and many have limited real-time performance when applied to realistically large data-sets: much of the user's cognitive capacity is therefore focused on controlling the software and manipulating esoteric file formats rather than on performing the research.

**Methods:**

To confront these issues, harnessing expertise in human-computer interaction (HCI), high-performance rendering and distributed systems, and guided by bioinformaticians and end-user biologists, we are building reusable software components that, together, create a toolkit that is both architecturally sound from a computing point of view, and addresses both user and developer requirements. Key to the system's usability is its direct exploitation of semantics, which, crucially, gives individual components knowledge of their own functionality and allows them to interoperate seamlessly, removing many of the existing barriers and bottlenecks from standard bioinformatics tasks.

**Results:**

The toolkit, named Utopia, is freely available from .

## Background

Biological databases and their associated bioinformatics analysis tools are widely used in modern biology. In the 1980s, when the tools and data repositories began to proliferate, researchers could not have foreseen how later users might ultimately want to consume and manipulate their data, or how they might want to connect related information derived either from different databases or from different analysis tools. When the numbers of available repositories and analysis tools were few, linking information between them was not a major concern. However, with the inexorable growth and the increasing number of bio-databases, which now total more than a thousand [1], finding the relevant resources and making connections between their contents and their analysis outputs pose significant challenges. There are numerous practical issues:

### Locality

A typical analysis task involves combining data from globally accessible 'primary databases' with locally held experiment-specific data, and manipulating the results using a combination of global/public and local/private tools. Industrial users are often forced, for security and privacy reasons, to take in-house copies of the primary databases and tools. The practical limitations of the uses of network, CPU and storage often mean that other users too end up taking local copies, and thus must also deal with maintaining and synchronising these with their remote counterparts.

### Architectural

Bioinformatics tools have a variety of architectures. Some are embedded in web pages as Java applets, or as is increasingly common, other dynamic content frameworks such as CGI, Ruby on Rails, Ajax, Struts, PHP or Python Pylons. Others are command-line tools written in a variety of programming and scripting languages, some of which are available in platform-specific executable form, whilst others require compilation or the installation of additional support libraries. Yet others are fully blown interactive applications with graphical or command-driven interfaces. Architectural integration between such components presents many pragmatic problems.

### Legal

Many licensing strategies have been adopted in the field, ranging from expensive per-tool-peruser-per-year bespoke agreements on one hand, to Open Source licences (of which 72 variants have been approved by the Open Source Initiative [2] alone) on the other. Combining tools with different licensing agreements can quickly become a legal nightmare, especially if the intention is to then make the new resource publicly available.

In addition, two more subtle issues confound progress:

### Usability

With their mix of architectures behind the scenes, tools and databases present users with a bewildering variety of user-interaction paradigms and visual styles. Many of the most popular and high-profile resources have interfaces that meet or approach modern best practice in design for usability, but even here there is inevitably considerable variation in style and functionality between providers. Many tools remain with interfaces that, while being optimal for their creator's original requirements, seem complex or esoteric when distributed to a wider community with more diverse needs. Learning to use all of these in concert is a substantial challenge for users.

### Semantic integrity

Perhaps the most complex and pernicious of these issues is that of semantic integrity: *i.e.*, that of interpreting the real meaning of data derived from multiple sources or manipulated by multiple tools. Even for relatively modest real-world scenarios, representing these concepts formally, even within a single database, presents significant challenges. Without such formalisation, however, the burden of interpretation, to determine the validity and meaning of the data, remains with human users.

Against this background, finding, establishing and maintaining connections between relevant tools and the most up-to-date data is hard, and consequently much user-effort is expended on mundane 'administrative' tasks rather than on novel science.

For example, consider the common analysis task of multiple sequence alignment. Numerous programs are available to visualise and/or create alignments: these include stand-alone automatic multiple alignment tools, accessible as command-line applications or via web forms (*e.g.*, ClustalW [3], T-Coffee [4], MUSCLE [5]); components of large (often commercial) integrated packages (*e.g.*, pileup in GCG [6]); command-line driven alignment editors with X-windows interfaces (*e.g.*, XALIGN [7]); manual editors written as Java applets or downloadable applications (*e.g.*, CINEMA [8-10], JalView [11]); or X-windows-based alignment viewers (*e.g.*, Belvu [12]). The multiplicity of tools is both confusing for end users and wasteful for developers, as many have not been developed with re-usability or extensibility in mind. To give a trivial example, most of these programs use different input and output formats: NBRF-PIR, FastA, Clustal, GDE, PHYLIP, MSF, to name but a few. Hence, even if a user knows how to find the tools he seeks, transforming the data output from one into a form that is intelligible to another is not straightforward. To remedy this, software has had to be developed whose sole purpose is to convert between the many formats in which biological sequences and their alignments are most typically held. From a user perspective, exporting an alignment from an automatic package into a manual editor therefore means the use of such a format-conversion program is likely to be necessary; from a developer perspective, integrating an automatic alignment tool into an existing manual editor (or vice versa) means that an appropriate format-exchange program must either be written, or bundled, into the system.

The current situation is therefore far from optimal, with hundreds of bioinformatics tools and databases scattered across the internet and local intranets. Before the data can be of use, much time and intellectual energy is wasted by users in finding the right database or databases, in understanding the differences between the types of information stored, in checking that the information stored is up-to-date, in locating the right analysis tool or tools necessary for the task, and ultimately, in making the necessary file transformations to convert data in different formats from the different tools and databases into forms that are tractable for their analyses. A testament to the difficulty of the problem is that routine bioinformatics tasks like text and sequence searches, sequence alignment, sequence property calculations, sequence annotation, three-dimensional (3D) structure visualisation, and so on, are still tediously slow to perform using standard tools, and the results of such tasks remain unintegrated. If we are to make progress in analysing the flood of biomolecular sequence and structure data that will in future continue to pour from high-throughput genomics and proteomics experiments, a general solution is needed that will make bioinformatics analyses as easy and intuitive to perform as using standard 'office' software. This is a challenge, given that biologists tend to work with significantly more complex data than are represented by a collection of word-processor documents and spread-sheets.

## Old problems, new perspectives and barriers to progress

Given the problems, we wanted to take a fresh look at how to provide bioinformatics tools and databases in a user-friendly and robust manner in the face of legal, architectural and topological issues. The work environment today is data-rich, and HCI and visualisation techniques have become pivotally important both for accessing those data and for manipulating them: our initial focus has therefore been on *usability*. The ideal bioinformatics working environment would be one in which a user's analytical abilities are actively *supported *rather than just *enabled *by computational tools, where the interface is clear, consistent and communicative, where users do not have to worry about underlying file-types, software architectures or operating systems, but can use whatever tools they need within a clear, visually supportive and intuitive framework. Within such a system, it would be helpful to be able to perform standard tasks (database and literature searching, sequence alignment, phylogenetic analysis, molecular visualisation, *etc.*), to be able to easily customise and extend its functionality, and to be able to access the most up-to-date versions of the necessary tools and resources with minimal effort. It would also be valuable if this idyllic system afforded a collaborative environment, allowing users in different locations to visualise and interact with the same data. This would be especially useful in projects based in different geographical locations, or in training or community-learning settings.

To make further progress, we need to be able to tackle these problems coherently. This is not easy, and various attempts have met with different success rates in the past. The following section looks at some of these endeavours and why they have fallen short of a general solution.

## The current scenario

Over the years, a number of solutions to these interoperability issues have been presented. These have tended to be characterised by the development of, on the one hand, integrated tool environments, and on the other, integrated database environments. However, such integrated environments are not without problems of their own: they are hard to maintain, they are hard to keep up-to-date, they are hard to extend or customise, they lose immediacy and support for interactive analysis tasks, and users can only perform tasks or use tools the designers predicted they would want to perform or use. Moreover, such environments tend to grow in complexity, with new functionality being added in a piecemeal way in response to user requests or developer whims, the resulting package ultimately requiring considerable effort from the user in battling with its front-end rather than in understanding its outputs.

A classic example of an integrated database environment is the unified protein family resource, Inter-Pro [13]. In the beginning, InterPro amalgamated four different protein signature databases: PROSITE [14], which houses regular expressions and profiles; PRINTS [15], which exploits position-specific scoring matrix-based fingerprints; Pfam [16], which uses hidden Markov models; and ProDom [17], which uses automatically-generated sequence clusters. The diagnostic methods exploited by these resources are different but complementary, providing different perspectives on protein family relationships.

Ten years ago, when the component databases were relatively small, integrating and rationalising their data was relatively straightforward; but as each resource has grown, the familial boundaries defined by their different approaches have been blurred, and the relationships between families have become more fuzzy. Over time, managing and representing these biological overlaps in a meaningful way for end users has consequently became a major challenge. Consider, for a moment, the entry for rhodopsin-like G protein-coupled receptors (IPR000276). According to InterPro, the superfamily contains 19898 members: of these, 19592 were identified by Pfam's hidden Markov model, 16868 by the PRINTS fingerprint and 16478 by the PROSITE regular expression. By contrast, the source databases themselves quote 16975, 1143 and 2029 members respectively. Clearly, these numbers are very different, and at least point to a synchronisation problem: InterPro tracks the latest version of UniProt, but the source databases lack the manpower to achieve this. Users are therefore left to work out the relationships between the family membership suggested by the source databases (16975, 1143, 2029) and that suggested by InterPro's implementation of the source database's diagnostic tools (19592, 16868, 16478), and the unified number endorsed by InterPro itself (19898), which is larger than the number identified by any of the component tools.

For end users, this kind of complexity has been exacerbated in recent years both by the addition of 6 further source databases and by the inclusion of links to more than 20 additional external resources. Bit by bit, the web interface had to be adapted to accommodate these changes, and to provide more and more new functionality to meet end-user needs: *e.g.*, with new protein-match and domain-architecture views, signature relationships and sequence coverage views, taxonomy browsers, structural links, ontology terms, literature cross-references, further reading, and so on. During the last decade, the interface to InterPro has consequently grown alarmingly complex, so much so that it has become necessary to run workshops to explain how to use the resource; fortunately, a major redesign of the interface is now underway [18].

These problems aside, such approaches are only partial solutions because the integration they offer is ultimately just an illusion: the systems themselves lack explicit semantics, so the burden remains on users to know what the tools are, what they do, how to use them, what options are available and how these work, whether the information provided is up-to-date, whether it is compatible with that held in source repositories, and so on. Such issues provide a strong motivation to develop more user-friendly approaches: users need to be able to access standard bioinformatics tools from an intuitive, integrated environment that exploits familiar interaction metaphors; they need protection from the technological intricacies of accessing heterogeneous resources, the complexities of which should be hidden behind the familiar desktop paradigm (drag-and-drop, cut-and-paste, *etc.*), without trivialising the problems of data integration and limiting the kind of functionality available. In short, they need access to interfaces that 'just work', so that they do not continue to waste cognitive effort battling with the technology but can actually get on with their research.

## Semantic integration

As already mentioned, the legal, architectural and topological heterogeneity of existing tools and databases makes traditional forms of integration exceptionally difficult: the huge numbers of licences, libraries and languages present significant challenges in terms of software engineering. Even when these difficulties can be overcome at a technical level, users and developers must take extreme care to ensure that the results of such integrated tools mean what they think they do.

The concept of Semantic Integration [19,20] comes originally from the world of business and electronic commerce, where similar problems of legacy software and complex data exist. The approach focuses on the use of metadata to describe the meaning of data, and the construction of ontologies that enable concepts in one system to be mapped to those in another, irrespective of the technology used to process or store those data. Here, we apply these ideas to the field of bioinformatics, where the problem is arguably more complex. In commerce, the concepts being manipulated are predominantly man-made, and their constituent parts are thus reasonably well defined; in biology, we are attempting to associate meaning and relationships with real-world phenomena that are only partially understood and are continuously unfolding. Nevertheless, integration by semantics promises significant advantages over more traditional approaches, both in terms of insulating components from technological incompatibilities, and by allowing more formal rigorous meaning to be associated with data.

## Methods

In recent years, we have been developing a user-centred, semantically integrated framework that brings modern visualisation, HCI and knowledge-management techniques to the problem of analysing bioinformatics data. Our approach involves a software architecture that explicitly models a hierarchy of semantics, starting, at its most abstract, with the User's Conceptual Model, and following this through progressively concrete levels to eventually match up with the (implicit) semantics and architectures of existing 3rd party tools. The software suite, named Utopia (after its founding project, UTOPIA – User-friendly Tools for OPerating Informatics Applications), consists of a set of user-friendly, interactive and interoperable graphical tools combined with seamless access to distributed workflows, web services and databases. The key aspect of Utopia is that semantics permeate the system in its entirety: from a field in a database to a pixel illuminated on the screen, the explicit use of semantics allows Utopia to reason about the data being manipulated and to modify its behaviour accordingly.

**Figure 1:**
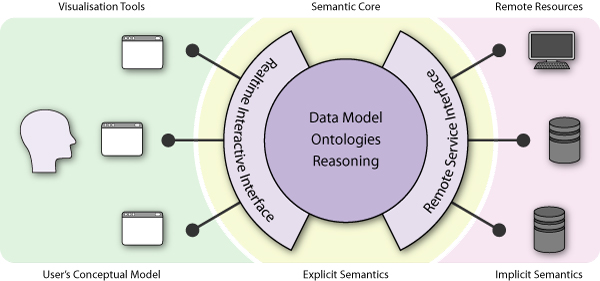
**The semantic architecture of Utopia**. The Utopia architecture consists broadly of three layers: the visualisation tools, which encapsulate the User's Conceptual Model; the core, which encodes and reasons over explicit semantics in its data model; and finally, the remote resources and third-party tools with their implicit semantics.

The system's architecture is broadly separated into three layers, as shown in figure 1. A user sees only the analysis and visualisation tools represented at the left of the figure. These are designed to provide intuitive access to their functionality, with an emphasis on usability 'best practice', and map the myriad data types to a smaller number of concepts in the User's Conceptual Model. They appear as independent applications, and indeed can even be used as such, but in fact they share their data with each other via the underlying core, providing similar interoperability to standard Office software. For example, data can, where appropriate, be dragged-and-dropped between tools, providing a coherent working environment for the user. Hidden from the user's view, and responsible for managing and marshaling data, is Utopia's semantic core: this takes care of data storage and access, initialisation, finalisation and provides the mechanisms of system extension. The core provides real-time access to data and is semantically agnostic, relying on the data sources and visualisation tools to explicitly specify meaning. The final layer consists of the system's pluggable extensions: format parsers, serialisers, bootstrapping functionality and, most importantly, generic *conduits *designed to loosely couple the system to external resources that provide data and algorithmic functionality via workflows and web services. Each layer explicitly allows for the modelling of domain semantics, matching the 'low-level' meaning of individual tools and data repositories to 'high-level' concepts represented in the user interfaces. The following sections describe the architectural components in more detail.

### The data model

At the core of the Utopia system, and key to its functionality, is a data model designed to be rich enough to capture the semantics of bioinformatics data in such a way that it can be exchanged between applications, and at the same time sufficiently light-weight to be interrogated in real-time to extract the objects required by the interactive visualisation tools [21]. These two goals are generally at odds with one another: the level of abstraction required by the model (if that model is to represent such a complex domain as biology) tends to equate to methods of data storage that are ill-suited to many of the strategies employed in complex real-time visualisation. Achieving both of these goals simultaneously therefore requires a novel solution.

#### Achieving real-time performance

Interactive visualisation of large data-sets is a common requirement in bioinformatics. In order to give the illusion of smooth movement (either through dynamic simulation or direct manipulation of the user interface), an application must be capable of visualising multiple distinct frames per second (fps). For 3D visualisation, which is perhaps the most intensive form of visualisation, it is generally desirable to achieve around 24 fps, depending on subject matter [22]. The difference in usability between a sluggish visualisation and a responsive one can be dramatic. With a reasonable frame-rate and suitable interaction paradigms, a user becomes *absorbed *or *immersed *in the content of the visualisation – *i.e.*, is able to concentrate on the task at hand and on understanding the data. Below an acceptable threshold, the user becomes increasingly aware of the intrusion of the tool itself into their mental processes, and cognitive capacity is wasted 'smoothing out' their experience. Speed of access is therefore crucial to interactive visualisation. Existing approaches to the kinds of abstract modelling required by Utopia, such as implementations of the Resource Description Framework (RDF), are optimised not for speed, but for flexibility, and random access. Utopia overcomes this problem by using a data structure with the expressivity of RDF, but taking into account in its implementation the most common types of spatial and temporal access strategies required by visualisation and analysis. For example, this means that sequential access to related data, and the resultant efficiency of such access, is built directly into the model, and as such takes precedence over the random access required by more abstract queries, such as those of the Simple Protocol and RDF Query Language (SPARQL) [23].

When looking at the requirements of many visualisation methods, stability of data structures is of key importance, and this is where our second conflict lies. For small models and brute-force visualisation strategies, it is perfectly acceptable for a data structure to be unstable throughout the lifetime of the application: if a representation is re-computed from scratch for every frame, the stability in memory of its model is irrelevant. However, many visualisation techniques (most especially those for rendering large models, or for rendering 3D representations of data) make use of optimisation strategies such as geometry compilation and bitmap caching; in these instances, a locally stable data structure is important to the effectiveness of those optimisations. Unfortunately, many approaches to abstract data storage make use of structures that are not locally stable. An example of this is the popular use of hash maps to store objects of interest: adding one new object could potentially (depending on the hashing function used and the previous state of the hash map) cause the map to be re-sized and re-organised in memory. This would almost certainly change the large-scale organisation of the objects in the model, and so would invalidate many visualisation strategies. The same approach that gives Utopia its fast access also solves the problem of local stability: multiple objects related to a subject according to the same property are implicitly ordered in its data structure; relatives are accessed in the order in which the relationships were created. Ordering can change dynamically, through explicit intervention by the user, but between such explicit restructuring tasks, and non-locally to changes, ordering is stable.

#### Encoding semantic spaces

As already mentioned, the Utopia model is designed to hold any arbitrarily complex data, in much the same way as does RDF. As a goal in itself, this has advantages, but it is the ability to encode explicit semantics that provides Utopia with its reasoning skills. Though homogeneous by implementation, the model's data structure can be conceptually split into orthogonal *spaces *such that these semantics can be seen as separate from the data and metadata to which they pertain.

First, a distinction is made between structure and annotation: *i.e.*, between concepts that are accepted as 'fundamental facts' within a domain, and concepts that augment or enrich the knowledge of the structure in some way but are in themselves either 'received wisdom', fuzzy, or refer to a process or collection of structural concepts. Unlike in the physical and mathematical sciences, where discoveries are axiom based, very few of the concepts in the biological domain can be thought of as absolute truths: beyond such things as atoms, bonds, residues and sequences, the majority of biological features contain degrees of uncertainty or ambiguity that must somehow be represented within the model in order that they can be rendered as visual objects. Utopia's *structure space *is therefore quite small, and consists of only a handful of types of node: bonds, atoms, residues and sequences being the most common in bioinformatics. All other concepts are mapped as annotations that project onto this structure space, and themselves comprise *annotation space*. Each annotation may map to a single node in structure space, or to a set of nodes. An annotation may also have associated provenance.

Most important is how each of the nodes of both the structure and annotation spaces are semantically tagged using terms from various (and extensible) ontologies that reside within the model's *semantic space*. This not only gives data and metadata meaning in a particular domain or context, but allows such classifica-tions to be inter-related and grouped in a hierarchy if appropriate. As an example of this, take the following ontological relationships expressed as triplets of subject, property and object:

(1)

(2)

(3)

From expression (1) we see a general class of annotations that pertain to contiguous extents along a protein sequence (for example); expressions (2) and (3) show two particular specialisations of this concept, those of transmembrane (TM) domains and secondary structure assignments, respectively. Even this trivial ontology fragment can afford Utopia the ability to reason over different classes of annotation, and provide tailored visualisations accordingly.

Finally, *variant space *represents uncertainty, conflict and alternatives within a data set. A variant node maps onto a set of structural nodes that all purport to represent the same data, and provides a mechanism for making any identifiable ambiguity or conflict explicit in the model.

The separation of these spaces allows their implementation to be tailored to their most common use within the core. At one extreme, for semantic integration, a certain amount of (heavyweight) computational reasoning may be required to infer that an 'enzyme' is-a-kind-of 'protein' so that it can be viewed in a sequence viewing tool. Thus, access to *semantic space *is typically optimised for RDF-style random access. However, the data structures and algorithms to support this reasoning must not interfere with the need to rapidly extract tens of thousands of objects that form a systems biology graph, or hundreds of thousands of atoms twenty-five times a second in order to be able to render a ribosomal complex as an interactive 3D structure. Hence, the underlying data structures that support the *structure space *are optimised to exploit the spatial and temporal coherency in order to provide rapid and stable access.

This underlying model, therefore, allows Utopia to gather and integrate data from a wide variety of heterogeneous sources and to generate a canonical internal representation that can be visualised by any of the front-end tools using semantic annotations to guide them. Of course, semantically annotated data is only part of the story: functionality of both tools and extensions are similarly annotated, explicitly describing the meaning behind their uses. Tools, then, negotiate with the core using terms from the semantic space (*e.g.*, 'can render sequences of residues with regional annotations', 'can show a fingerprint motif' or 'can display a structure of atoms with regional annotations') and thus do not have to be aware of file formats or the means of accessing remote sources of data. The richness of the model has two additional important features: (i) multiple Utopia tools are inherently aware that they are viewing the same biological concept, albeit potentially in radically different forms (*e.g.*, as a sequence of residues, as a molecular structure, or as a frequency plot), and hence modifications made to the data in real time by one tool and injected into the underlying model are immediately reflected in any other; and (ii) biological concepts are exposed as 'first class citizens' in the interface itself, and hence the tools are aware that the user has selected 'a sequence', 'an alignment of sequences', 'a signalling pathway', a 'cell compartment' and so on.

### Access to remote resources

Utopia's graphical tools allow the user to interactively manipulate and view biological concepts. Of themselves, they do not provide any algorithmic functionality, which is instead provided by 3rd-party tools and services. These can be in the form of locally installed programs, or more commonly, web services or work-flows [24].

The system is able to make use of such remote resources through its extension mechanism. Generic plugins, or *conduits*, provide the semantic descriptions neccessary for a tool to communicate with the core, and handle any low-level data manipulation or architectural features necessary for integration. The semantic annotations provided within the conduit give Utopia two key advantages: abstraction of file formats, and reasoning over functionality.

The abstraction is achieved by the core, and the visualisation tools that use it, communicating in coherent high-level semantic concepts, with each conduit individually dealing with the low-level pragmatic issues of file formats and so on. The semantic annotations provided by each conduit also allow the visualisation tools to modify their user interfaces to accommodate new functionality on the fly, providing task and context sensitive behaviour.

**Figure 2:**
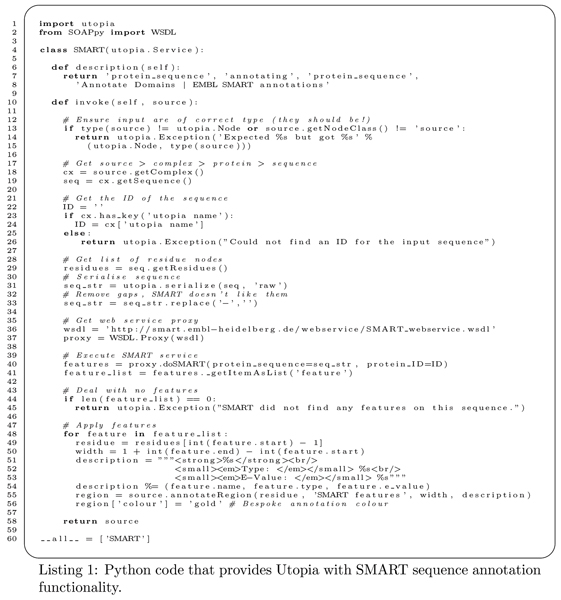
**SMART conduit listing**. The Python code listing of the conduit for providing Utopia with the analysis functionality of the SMART (Simple Modular Architecture Research Tool [29]) web service. The code defines a single Python class, with a description method that provides terms from an ontology defining the inputs, function and outputs of the conduit, as well as a human readable comment. The remainder of the class is taken up by the invoke method that extracts data from the semantic model, accesses the SMART web service, decodes the results, and finally modifies the semantic model before returning.

Plugins are simple to write but are nevertheless very powerful: currently, they can be coded in C++ or, more commonly, Python. The Python excerpt illustrated in figure 2 uses the SMART web service [25]) to annotate a protein sequence and return it to Utopia. The 'description' method at the head of the code advertises this plugin's functionality to Utopia using terms from its ontology, which allows Utopia to deduce where to put this feature in its interface, when to display it to the user (in this case, in any context menu where 'annotating' an object of type 'protein sequence' would be useful), and what inputs and outputs are required to invoke the service. A full tutorial on writing plugins is available from the Utopia website .

### The visualisation tools

**Figure 3:**
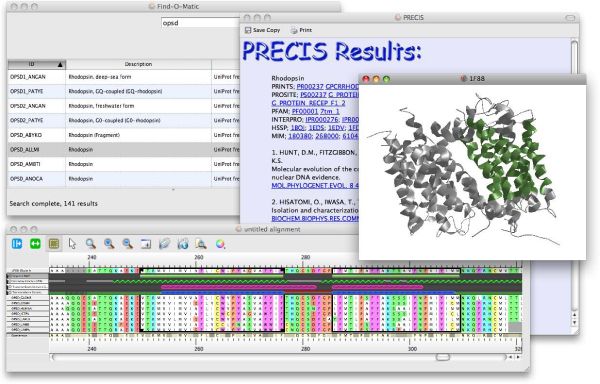
**The Utopia suite**. Screenshot of selected tools from the Utopia suite, as used in the Case Study: top left shows Utopia's search tool, having located a number of rhodopsin sequences in UniProt; the bottom image illustrates the Sequences alignment editor, in which sequences selected from the search results have been automatically aligned – highlighted are two manually-selected TM domains (denoted by the green bars), contrasting the results from two TM-prediction tools (denoted by the pink and blue hatched bars) with the known locations of the TM helices from the crystal structure of bovine rhodopsin (green zigzags); centre right shows the Structures tool, depicting the 3D structure of rhodopsin, with the manually-selected TM domains highlighted in green; beneath this is shown the PRECIS report, providing relevant literature citations, database cross references, details of the protein function, disease associations, and so on. Used in this way, the efficacy of the TM prediction tools relative to the known structure is striking.

**Figure 4:**
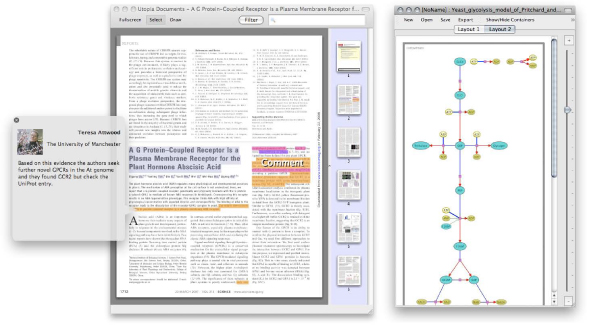
**Forthcoming tools**. Screenshots of Utopia Documents (left) and Networks (right). In Documents, the different coloured highlights represent areas of interest, determined algorithmically or by manual annotation (for example, here the comment bubble shows a reader adding a commentary to this paper, which is then automatically shared with all other readers). In Networks, nodes are coloured according to whether they represent metabolites or their co-factors.

The released version of the Utopia suite currently has three front-end applications: Sequences, Structures and Library; with two further tools in development: Networks and Documents. These are illustrated in figures 3 and 4.

**Sequences **is a fully-featured sequence alignment editor. Alignments can be visualised at different scales: from close-up, suitable for detailed editing tasks, through to a pixel-per-residue overview. Multiple views can be open simultaneously, allowing different regions to be shown at the same time, or for the same region to be compared at different levels of detail, using separate colour schemes if desired. Beyond the sequence data presented by traditional alignment editors, Sequences is able to relate other associated information to the sequences and display this in a suitable graphical format by exploiting Utopia's semantic data model. For example, annotations relating to individual residues can be drawn as pointers below those residues; annotations representing contiguous regions, such as conserved motifs or TM domains, can be drawn as coloured bars below the appropriate section of sequence; and those consisting of continuous values associated with individual residues can be drawn as a graph or spectrum of colours. Forthcoming enhancements to Sequences will enable DNA and RNA sequences to be seamlessly viewed and manipulated alongside their protein products.

**Structures **is Utopia's 3D macromolecular structure viewer. It exploits modern Graphical Processing Unit (GPU) techniques to accelerate high-quality, high-fidelity rendering of very large molecular models in real time. Structures currently supports a number of representation styles, including 'space fill', 'backbone', and 'cartoon' rendering, and is able to overlay annotations from the semantic model on all of these. In the near future, we plan to extend Structures to display small molecules in 2D and 3D for use in biochemical scenarios.

**Library **provides an iTunes-like interface for discovering services and data objects. Simple keyword-based queries are submitted to multiple databases and tools, results being returned in a unified format that can be sorted, arranged into 'playlists', and moved interactively to the other visualisation tools. Modern data-management techniques, such as tagging and real-time filtering, are also included.

In addition to these, two other tools are in preparation:

**Networks **is a tool for visualising graph-like relationships between objects, for example protein-to-protein interactions, metabolic pathways or 'mind maps'. While many generic and biology-specific graph-layout tools exist, Utopia Networks focuses on i) creating intelligible domain-specific diagrams by matching familiar drawing styles with layout algorithms driven by hints gleaned from the underlying semantics stored in the model; and ii) on inter-relating objects in the graph with their representation in other Utopia tools – *e.g.*, selecting a metabolite in a pathway allows its structure (and, if appropriate, its sequence) to be examined and manipulated. Figure 4 (right hand side) illustrates Utopia Networks visualising the Pritchard and Kell Yeast Glycolysis model [26].

**Documents **extends Utopia's domain beyond biological databases and tools to the scientific literature in general. Ostensibly a PDF viewing tool, Documents enables references to biological objects in academic articles to be linked dynamically to their counterparts in databases, both by authors and readers. Tight integration between the suite's tools will allow static illustrations of sequences, structures and networks in papers to be associated with up-to-date interactive equivalents. Figure 4 (left hand side) shows Utopia Documents viewing an annotated journal article: highlighted regions represent either bibliometric data automatically identified by the software (*e.g. *title and authors) or annotations added manually (*e.g. *database cross references, other hyperlinks, and user comments).

## Results

As a case study, we performed a series of routine bioinformatics tasks, which included the following: i) select a protein sequence from a database; ii) search for related sequences using BLAST; iii) select 10 sequences from the output for further study, including one of known structure; iv) produce an annotation report for the selected family of sequences using PRECIS [27]; v) align them using ClustalW or MUSCLE; vi) visualise the sequence of known structure in 3D; vii) visualise regions of this sequence annotated, say, as TM domains; viii) from the alignment, select a set of conserved motifs; ix) establish the 3D locations of the selected motifs; and x) compare the locations of predicted TM domains with known locations of alpha-helices in the 3D structure.

In tackling these tasks, the traditional approach requires the use of a combination of local and remote resources, and often manual transfer of data between them: specifically, the above scenario requires the use of six separate tools, each with different interfaces (and a variety of input and output requirements), and a similar number of manual data-translation tasks. By contrast, the Utopia approach combines all of this functionality within the same suite (as illustrated in Figure 3) by calling on remote Web services, obviating the need for users to interact with file formats or to worry about input/output parameters. From a usability perspective, therefore, Utopia provides a more efficient means of achieving the same goal, keeping the scientific problem in focus and reducing the number of technological distractions.

## Discussion

The Utopia approach differs from other integrative endeavours in that it directly supports semantically rich visualisations, an aspect of analysis often left to separate distinct tools. The main consequence of this is that, in order to integrate the functionality of remote resources, new plugins must be written for each to inject the necessary semantics into the model. Although Utopia's extension framework makes this relatively painless, providing access to apprioriate parsers and serialisers and a simple API to the model, it contrasts with other *open world *systems, such as Taverna's workflow engine, which can access resources with little more than a Web Service Description Language (WSDL) definition. Fortunately, this perceived disadvantage is alleviated by emerging standard mechanisms of access, such as the DAS specification, where both transport and semantics are uniform across all resources.

Another hurdle applies to the development of visualisation tools, which, if they are to make full use of Utopia's semantic core, must be capable of communicating bidirectionally with the model: access and visualisation should be accompanied by annotation and manipulation. Such complex tools are uncommon, so Utopia's main visualisation applications have had to be developed specifically.

Perhaps the largest inconvenience arises from the use of remote functionality over which neither users nor the system itself have direct control. Utopia is reliant on the correct execution of other institutions' web services: bugs in implementation, system outages, architecture changes, migrations, network load, bandwidth, firewalls, *etc.*, can each prevent that functionality from working correctly (or at all). This drawback aflicts *all *systems that rely on web services, and is not directly soluble with contemporary stateless protocols.

Fortunately, indirect solutions are possible, such as redundancy and automatic fallback. One initiative that endeavours to tackle these issues systematically is the EMBRACE Web Service Registry [28]. The registry tracks available web services and regularly tests their functionality to ensure both that results are produced, and that they are correct. Interestingly, although the registry has only been running a month, it has already highlighted a number of instabilities with web services that have otherwise gone unnoticed without such regular programmatic tests. It is planned for Utopia to be able to programmatically query these test results to alert users to service outages, and to suggest alternatives.

## Conclusion

The sub-fields of bioinformatics are now sufficiently mature to require a more integrated approach to data storage, management and visualisation. While Utopia is not the only solution to the current problems in bioinformatics, nor a panacea for all data-interoperability issues, it is a real working solution that offers easy-to-use tools for biologists and bioinformaticians. It achieves this by providing a data model that can represent the semantic information within the data; by providing end-user tools that are seamlessly interoperable; and by providing an extensible architecture that allows bespoke data sources and services to be added by users themselves.

## List of abbreviations used

3D: Three Dimensional; API: Application Programmer Interface; GPU: Graphical Processing Unit; HCI: Human-Computer Interaction; RCSB: Research Collaboratory for Structural Bioinformatics; RDF: Resource Description Framework; SMART: Simple Modular Architecture Research Tool; SPARQL: Simple Protocol and RDF Query Language; TM: Transmembrane; WSDL: Web Service Description Language.

## Competing interests

The authors declare that they have no competing interests.

## Authors' contributions

All authors have contributed equally to the conception, design and implementation of this system. All authors read and approved the final manuscript.
